# Sleep quality, restless legs syndrome and daytime sleepiness in adults with 5q-spinal muscular atrophy

**DOI:** 10.1177/22143602251400502

**Published:** 2025-12-16

**Authors:** Bogdan Bjelica, Kaan Bacara, Camilla Wohnrade, Alma Osmanovic, Olivia Schreiber Katz, Susanne Petri

**Affiliations:** 1 Department of Neurology, 9177 Hannover Medical School, Hannover, Germany; 2 PRACTIS Clinician Scientist Program, Dean's Office for Academic Career Development, 9177 Hannover Medical School, Hannover, Germany; 3 Essen Center for Rare Diseases (EZSE), 39081 University Hospital Essen, Essen, Germany

**Keywords:** spinal muscular atrophy, sleep quality, excessive daytime sleepiness, quality of life

## Abstract

**Background:**

This study aimed to investigate sleep quality, restless legs syndrome (RLS), and excessive daytime sleepiness in adults with spinal muscular atrophy (SMA) and their relationships with motor function, quality of life (QoL), fatigue, and depression.

**Methods:**

We included 43 adults with SMA (31 non-ambulatory; 11 using non-invasive ventilation) and 43 age- and sex-matched healthy controls (HC). Subjective sleep quality and daytime sleepiness were assessed using the Pittsburgh Sleep Quality Index (PSQI) and the Epworth Sleepiness Scale (ESS). RLS was diagnosed using the International RLS Study Group diagnostic criteria. Revised Upper Limb Module (RULM), Hammersmith Functional Motor Scale Expanded (HFMSE) and respiratory function, as well as, 36-Item Short Form Health Survey (SF-36), Beck Depression Inventory (BDI) and Fatigue Severity Scale (FSS) were recorded.

**Results:**

SMA patients had significantly worse scores in sleep efficiency domain of the PSQI (0.7 ± 1.0 vs. 0.3 ± 0.6, p = 0.04) as well as in global PSQI score compared to HC (5.5 ± 3.5 vs. 4.0 ± 2.6, p = 0.029). Patients classified as poor sleepers (PSQI > 5) had a higher body mass index (BMI), greater BDI, and ESS scores. No association between global PSQI score and HFMSE or RULM score was observed. Patients classified as poor sleepers had lower total SF-36 score in comparison to good sleepers (53.2 ± 15.4 vs. 69.6 ± 12.3, p < 0.001). RLS was present in five SMA patients (11.6%).

**Conclusion:**

Poor sleep quality is common in adults with SMA. It contributes to a lower QoL and should be addressed as a part of the standard of care in adults with SMA.

## Introduction

Spinal muscular atrophy (SMA) is a rare, progressive, autosomal-recessive disease affecting lower motor neurons.^1^ It results from a deficiency of survival motor neuron (SMN) protein due to homozygous deletions or mutations in the *SMN1* gene located on chromosome 5q13.2. SMA is characterized by progressive muscle weakness, atrophy, and, in advanced stages, bulbar and respiratory insufficiency. The disease is classified into five types based on the age of onset and motor milestones achieved, ranging from SMA type 0, which manifests *in utero*, to SMA type 4, which presents in adulthood with relatively mild symptoms.^1^ For adult SMA patients, two disease-modifying therapies are currently approved: nusinersen, an intrathecally administered antisense oligonucleotide, and risdiplam, an orally available small molecule. Both treatments increase SMN protein production by enhancing *SMN2* gene expression.^2^

Sleep-related complaints are well-recognized yet often underdiagnosed complications of neuromuscular diseases, significantly contributing to morbidity and mortality.^3^ Given that there is a close relationship between sleep quality and quality of life (QoL) in the general population,^4^ early identification and management of impaired sleep quality in SMA are crucial. While motor function scales such as the Hammersmith Functional Motor Scale Expanded (HFMSE) and Revised Upper Limb Module (RULM) are essential for quantitative assessment of disease severity and progression, other factors such as the ability to perform daily activities, fatigue, endurance, bulbar function, and sleep quality are equally critical for overall patient well-being.^5–7^ A qualitative study by Lemus de et al. emphasized the importance of sleep and rest in evaluating the burden of SMA.^5^

To date, only a few studies have examined subjective sleep quality and excessive daytime sleepiness in individuals with SMA. A study in children with SMA type 2 and 3 using the Sleep Disturbance Scale for Children reported abnormal sleep scores in 16.4% of participants, with an additional 16.7% showing impairments in at least one sleep factor, though no correlation with functional scores was found.^8^ In contrast, a study in adults with SMA type 3 treated with nusinersen found no significant differences in sleep quality nor excessive daytime sleepiness compared to controls but did identify associations with functional scores.^9^ In a study by Crescimanno et al. half of the included adolescent and adult SMA patients were classified as poor sleepers, with sniff nasal inspiratory pressure emerging as the only independent predictor of sleep quality in their cohort of SMA type 2 and 3 patients.^10^ Despite the well-established links between sleep quality, QoL, fatigue, and depression,^4^ they have not been systematically investigated in adults with SMA to date. Furthermore, no study has yet investigated sleep quality longitudinally in this population.

Restless legs syndrome (RLS) is a common neurological sleep-related movement disorder that manifests as an uncontrollable urge to move the legs, typically accompanied by unpleasant or uncomfortable sensation.^11^ While RLS has been studied in amyotrophic lateral sclerosis (ALS), another motor neuron disorder,^12^ its prevalence and clinical features in patients with SMA remain unexplored to date.

This study aimed to evaluate overall sleep quality, excessive daytime sleepiness and RLS in adults with SMA in comparison to a healthy control group, examine its relationship with motor and respiratory function, as well as with QoL, fatigue, and depression and provide longitudinal data on sleep quality in adults with SMA.

## Methods

In this monocentric study, we included all adult SMA patients treated in both inpatient and outpatient settings at the Department of Neurology, Hannover Medical School. All SMA patients were 18 years or older and had a genetically confirmed diagnosis of 5q-SMA, defined by either a homozygous deletion of exon 7 or/and exon 8 of the *SMN1* gene, or a compound heterozygous deletion of these exons along with a point mutation in the second gene copy. The total SMA cohort comprised 43 patients, all of whom had a stable disease course and no symptoms or signs of any acute illness at the time of testing. Forty-three age- and sex-matched healthy controls (HC) were recruited from the authors’ immediate environment. To be enrolled, HC were required to be free of relevant medical conditions and to have no known history of insomnia, excessive daytime sleepiness, or sleep-disordered breathing. The study was approved by the Ethics Committee of Hannover Medical School (no. 6269), and all participants provided written informed consent.

We collected sociodemographic and clinical data from SMA patients, including sex, age, disease duration, SMA type, *SMN2* gene copy number, ambulatory status (defined as the ability to walk at least 10 meters without assistance or the use of mobility devices such as a cane or walker^13^), therapy at the time of testing, presence of scoliosis, use of non-invasive ventilation (NIV), and presence of percutaneous endoscopic gastrostomy (PEG), from patients’ medical histories. Participants’ weight status was determined based on their body mass index (BMI), calculated by dividing body mass (in kilograms) by the square of height (in meters). BMI categories were as follows: underweight (BMI <18.5), normal weight (BMI 18.5–25), overweight (BMI 25–30), and obese (BMI >30). Patients’ height was self-reported, while weight was measured using a calibrated scale. Participants in the HC group self-reported their height and weight. All assessments were performed during the same visit.

### Functional assessment

Motor function was assessed by trained physiotherapists using two widely applied scales: the RULM^14^ and the HFMSE.^15^ The RULM evaluates upper limb motor function and the ability to perform daily activities, consisting of 20 items with a maximum score of 37 points, where higher scores reflect better upper limb function. The HFMSE is a 33-item disease-specific scale that focuses on gross motor function, with a maximum score of 66 points, where higher scores indicate better motor abilities. Additionally, the Six-Minute Walk Test, which measures the distance a patient can walk in six minutes on a flat, hard surface, was administered to ambulatory SMA patients.^16^

Functional impairment was further assessed using the Spinal Muscular Atrophy Functional Rating Scale (SMAFRS) and the ALS Functional Rating Scale–Revised (ALSFRS-R).^17^ The SMAFRS is a disease-specific tool that evaluates functional abilities across 10 key domains of daily living. Each domain is scored from zero (fully dependent) to five (fully independent), with a maximum total score of 50.^18^ The ALSFRS-R comprises 12 items covering bulbar, fine motor, gross motor, and respiratory functions, each rated from zero (unable to perform) to four (normal ability), yielding a total score of up to 48, where higher scores indicate better function.

### Respiratory function assessment

Spirometry was performed in a seated position by a trained examiner using a calibrated Ganshorn Medizin Electronic BodyScope^®^ device. Patients with contraindications, such as respiratory infections, were excluded. Nose clips were used during the test. After two to three normal breaths, patients were instructed to perform a maximal slow exhalation, followed by a rapid maximal inspiration and expiration. Forced vital capacity (FVC), forced expiratory volume in one second (FEV1), and peak expiratory flow (PEF) were measured, and reference values of Global Lung Function Initiative were used.^19^ Three attempts were made, and if the results of the three attempts differed by more than 10%, the spirometry was repeated.

### Sleep quality assessment

Sleep quality was evaluated using the German version of the Pittsburgh Sleep Quality Index (PSQI),^20^ a self-reported questionnaire consisting of 19 items. It assesses seven key components of sleep quality: subjective sleep quality, sleep latency, sleep duration, sleep efficiency, sleep disturbances, use of sleep medication, and daytime dysfunction. Each component is scored from 0 to 3, and their sum produces a global PSQI score ranging from 0 to 21. A global score greater than 5 indicates poor sleep quality. Sleep quality was reassessed after one year in 30 SMA patients (10 patients declined to participate in the follow-up, and 3 questionnaires were excluded due to incomplete or incorrect completion).

### Excessive daytime sleepiness assessment

Excessive daytime sleepiness was assessed using the German version of the Epworth Sleepiness Scale (ESS),^21^ a self-reported questionnaire that evaluates the likelihood of dozing off in eight common daily situations. Each item is rated on a four-point scale (0 = no chance to 3 = high chance), with a total score ranging from 0 to 24. A score above 10 indicates increased daytime sleepiness. Excessive daytime sleepiness was reassessed after one year in 30 SMA patients (10 patients declined to participate in the follow-up, and 3 questionnaires were excluded due to incomplete or incorrect completion).

### Assessment of restless legs syndrome (RLS)

Presence of RLS was assessed by an experienced interviewer using the International Restless Legs Syndrome Study Group criteria (urge to move, worsening of symptoms at rest, relief with movement, symptoms at night)^22^ and RLS symptom severity using the German version of the International Restless Legs Syndrome Severity Scale (IRLS-SS).^23,24^ The IRLS-SS comprises 10 questions, each rated from zero to four, resulting in a total score range of zero to 40, where higher scores indicate more severe RLS symptoms. Patients with comorbidities that could mimic or contribute to RLS (such as iron deficiency, chronic kidney disease, diabetes mellitus, or autoimmune disorders) were excluded from this part of the study.

### Quality of life, depression and fatigue assessment

To assess health-related QoL, we used the German version of the SF-36 questionnaire,^21^ a widely applied generic tool that evaluates eight key aspects of health: Physical Functioning (PF), Role-Physical (RP), Bodily Pain (BP), General Health (GH), Vitality (VT), Social Functioning (SF), Role-Emotional (RE), and Mental Health (MH). These domains are further summarized into two overarching scores—the Physical Component Score (PCS) and the Mental Component Score (MCS)—alongside the total SF-36 score. Each domain is rated on a scale from 0 to 100, with higher scores reflecting better QoL.^25^

Depression was assessed using the German version of the Beck Depression Inventory (BDI), a self-reported questionnaire designed to measure the severity of depressive symptoms. It consists of 21 items, each scored from 0 to 3. A total score of 11 or higher was considered indicative of depression.^26^

Fatigue presence and severity were evaluated using the German version of the Krupp's Fatigue Severity Scale (FSS),^27^ a self-reported questionnaire consisting of nine items. It assesses fatigue intensity over the past week, with higher scores indicating greater fatigue. A total score of 36 or higher was used to define the presence of fatigue

### Statistical analysis

Statistical analysis was conducted using IBM^®^ SPSS^®^ Statistics (version 28, Chicago, IL, USA). Data normality was assessed with the Shapiro-Wilk and Kolmogorov-Smirnov tests, along with graphical inspection of histograms. Continuous variables were reported as median and interquartile range (IQR) (or minimum and maximum) or as mean and standard deviation (SD), depending on data distribution. Group comparisons were performed using the χ² test, Fisher's exact test, Mann-Whitney U test, or Student's t-test, as appropriate. A p-value <0.05 was considered statistically significant, while p < 0.01 indicated high statistical significance.

## Results

### Participants’ characteristics

Most SMA patients had SMA type 2 (46.5%) or type 3 (48.8%) and were non-ambulatory (72.1%). The majority were receiving either nusinersen (60.5%) or risdiplam (32.5%), while three patients (7.0%) did not receive any disease-modifying therapy at the moment of testing. Only three SMA patients were smokers. Use of antidepressants was reported in three patients, all of whom were treated with selective serotonin reuptake inhibitors (SSRIs). No statistically significant difference in BMI was observed between SMA patients and age- and sex-matched HC (p > 0.05).

The main sociodemographic and clinical data of the included SMA patients, as well as age- and sex-matched HC, are shown in Table 1.

**Table 1.** Main sociodemographic and clinical characteristics of adult SMA patients and healthy controls.

Features	SMA patients	HC
N	43	43
Male sex (N, (%))	23 (53.5)	22 (51.2)
Age at testing		
(years, mean ± SD)	37.6 ± 14.1	37.3 ± 14.1
(years, median [min-max])	37.0 [18.0–68.0]	37.0 [18.0–67.0]
BMI		
(kg/m^2^, mean ± SD)	23.1 ± 7.6	25.4 ± 4.7
(kg/m^2^, median [min-max])	22.6 [9.0–48.4]	24.5 [17.8–40.3]
Underweight (N, (%))	9 (20.9)	1 (2.3)
Normal (N, (%))	19 (44.2)	24 (55.8)
Overweight (N, (%))	9 (20.9)	12 (27.9)
Obese (N, (%))	6 (14.0)	6 (14.0)
Disease duration		-
(years, mean ± SD)	31.4 ± 13.8	
(years, median [min-max])	31.0 [5.0–66.5]	
SMA type (N, (%))		-
Type 1	1 (2.3)	
Type 2	20 (46.5)	
Type 3	21 (48.8)	
Type 4	1 (2.3)	
Therapy at the moment of testing (N, (%))		-
Nusinersen	26 (60.5)	
Risdiplam	14 (32.5)	
No therapy	3 (7.0)	
*SMN2* copy number		-
(mean ± SD)	3.5 ± 0.8	
(median [min-max])	3.0 [2.0–6.0]	
*SMN2* copy number (N, (%))		-
<4	24 (55.8)	
≥4	17 (39.5)	
missing	2 (4.7)	
Walking ability (N, (%))		-
Ambulatory	12 (27.9)	
Non-ambulatory	31 (72.1)	
Respiratory function		-
FVC		
(l, mean ± SD)	2.9 ± 1.6	
(l, median [min-max])	3.2 [0.2–5.7]	
FEV1		
(l, mean ± SD)	2.5 ± 1.3	
(l, median [min-max])	2.9 [0.2–5.0]	
PEF		
(l/s, mean ± SD)	5.7 ± 3.2	
(l/s, median [min-max])	6.0 [0.2–10.9]	
Scoliosis (N, (%))	33 (76.7)	-
Spondylodesis (N, (% out of all patients with scoliosis))	12 (36.4)	-
NIV (N, (%))	11 (25.6)	-
PEG (N, (%))	2 (4.7)	-
HFMSE score		-
(mean ± SD)	18.1 ± 21.8	
(median [min-max])	5.0 [0.0–63.0]	
RULM score		-
(mean ± SD)	19.3 ± 12.0	
(median [min-max])	17.0 [0.0–37.0]	
6-min walk test distance		-
(m, mean ± SD)	371.1 ± 162.6	
(m, median [min-max])	375.0 [25.0–575.0]	
SMAFRS score		-
(mean ± SD)	18.7 ± 18.1	
(median [min-max])	9.0 [0.0–50.0]	
ALSFRS-R score		-
(mean ± SD)	32.0 ± 8.5	
(median [min-max])	30.0 [14.0–48.0]	
BDI score		-
(mean ± SD)	4.6 ± 4.6	
(median [min-max])	4.0 [0.0–21.0]	
N (% of depressed)	10 (24.4)	
FSS score		-
(mean ± SD)	42.6 ± 12.1	
(median [min-max])	45.0 [12.0–61.0]	
N (% of fatigued)	29 (70.7)	
Total SF-36 score		-
(mean ± SD)	60.4 ± 16.2	
(median [min-max])	61.1 [15.9–91.6]	
Presence of RLS (N, (%))	5 (11.6)	-
IRLS-SS score		
(mean ± SD)	10.8 ± 6.7	
(median [min-max])	9.0 [4.0–22.0]	

SMA – spinal muscular atrophy; HC – healthy controls; N – number; SD – standard deviation; BMI – body mass index; *SMN2* – survival of motor neuron 2 gene; FVC – forced vital capacity; FEV1 – forced expiratory volume in the first second; PEF – peak expiratory flow; NIV – non-invasive ventilation; PEG – percutaneous endoscopic gastrostomy; HFMSE – Hammersmith Functional Motor Scale Expanded; RULM – Revised Upper Limb Module; m – meter; SMAFRS – Spinal Muscular Atrophy Functional Rating Scale; ALSFRS-R – Revised Amyotrophic Lateral Sclerosis Functional Rating Scale; BDI – Beck Depression Inventory; FSS – Fatigue Severity Scale; SF-36–36-Item Short Form Survey; RLS – restless legs syndrome; IRLS-SS – International Restless Legs Syndrome Severity Scale.

RLS was present in five SMA patients (11.6%), with a mean IRLS-SS score of 10.8 ± 6.7. Among these, three had SMA type 3, three had fewer than four *SMN2* gene copies, and three were female. Three patients were treated with risdiplam, four had scoliosis, and two had comorbid depression. Only one patient reported excessive daytime sleepiness, three were classified as poor sleepers, and none reported fatigue. No further statistical analysis was performed due to the small sample size of SMA patients with RLS.

### Comparison of sleep quality and excessive daytime sleepiness between adult SMA patients and heathy controls

SMA patients had significantly worse scores in sleep efficiency domain of the PSQI (0.7 ± 1.0 vs. 0.3 ± 0.6, p = 0.04) as well as in global PSQI score compared to HC (5.5 ± 3.5 vs. 4.0 ± 2.6, p = 0.029) (Table 2). Overall, SMA patients were more frequently classified as poor sleepers (as reflected by a PSQI score > 5) (55.8% vs. 34.9), although this difference did not reach statistical significance (p = 0.083). No significant differences were observed in mean ESS score between SMA patients and HC (p > 0.05).

**Table 2.** Comparison of sleep quality and excessive daytime sleepiness between adult SMA patients and healthy controls.

	SMA patients (n = 43)	HC (n = 43)	p value
PSQI components (mean ± SD)			
subjective sleep quality	1.0 ± 0.6	0.7 ± 0.6	0.064
sleep latency	1.1 ± 1.0	0.7 ± 0.7	0.060
sleep duration	0.3 ± 0.6	0.5 ± 0.7	0.212
sleep efficiency	0.7 ± 1.0	0.3 ± 0.6	**0.040**
sleep disturbance	1.0 ± 0.5	0.9 ± 0.5	0.127
sleep medication	0.0 ± 0.3	0.1 ± 0.3	0.704
daytime dysfunction	0.6 ± 0.8	0.2 ± 0.5	0.123
global PSQI score	5.5 ± 3.5	4.0 ± 2.6	**0.029**
good sleep quality (n, %)	19, 44.2	28, 65.1	0.083
poor sleep quality (n, %)	24, 55.8	15, 34.9	
ESS total score (mean ± SD)	4.7 ± 4.5	6.2 ± 2.9	0.057
score of <10 (n, %)	41, 95.3	40, 93.0	1.000
score of ≥10 (n, %)	2, 4.7	3, 7.0	

SMA – spinal muscular atrophy; HC – healthy controls; n – number; SD – standard deviation; PSQI – Pittsburgh Sleep Quality Inventory; ESS – Epworth Sleepiness Scale.

### Factors associated with poor sleep quality in adult SMA patients

#### Association of sociodemographic and clinical characteristics with poor sleep quality

SMA patients classified as poor sleepers (as reflected by a PSQI score > 5) had a higher BMI (20.3 ± 5.5 vs. 25.3 ± 8.4, p = 0.029), higher BDI scores (10.3 ± 9.4 vs. 3.9 ± 4.7, p = 0.012), and ESS score (6.0 ± 5.1 vs. 3.0 ± 3.1, p = 0.031), compared to SMA patients with good sleep quality. We observed no differences in mean global PSQI score in subgroup analysis regarding sex, current therapy, SMA type (2 vs. 3), ambulatory status, presence of scoliosis or spondylodesis, and use of NIV. Furthermore, no significant associations were found between global PSQI score and the following variables: age at testing, disease duration, *SMN2* gene copy number, respiratory function, HFMSE, RULM, ALSFRS-R, SMAFRS, 6-min walk test, or FSS.

#### Association of quality of life and its domains with poor sleep quality

**Figure 1.**
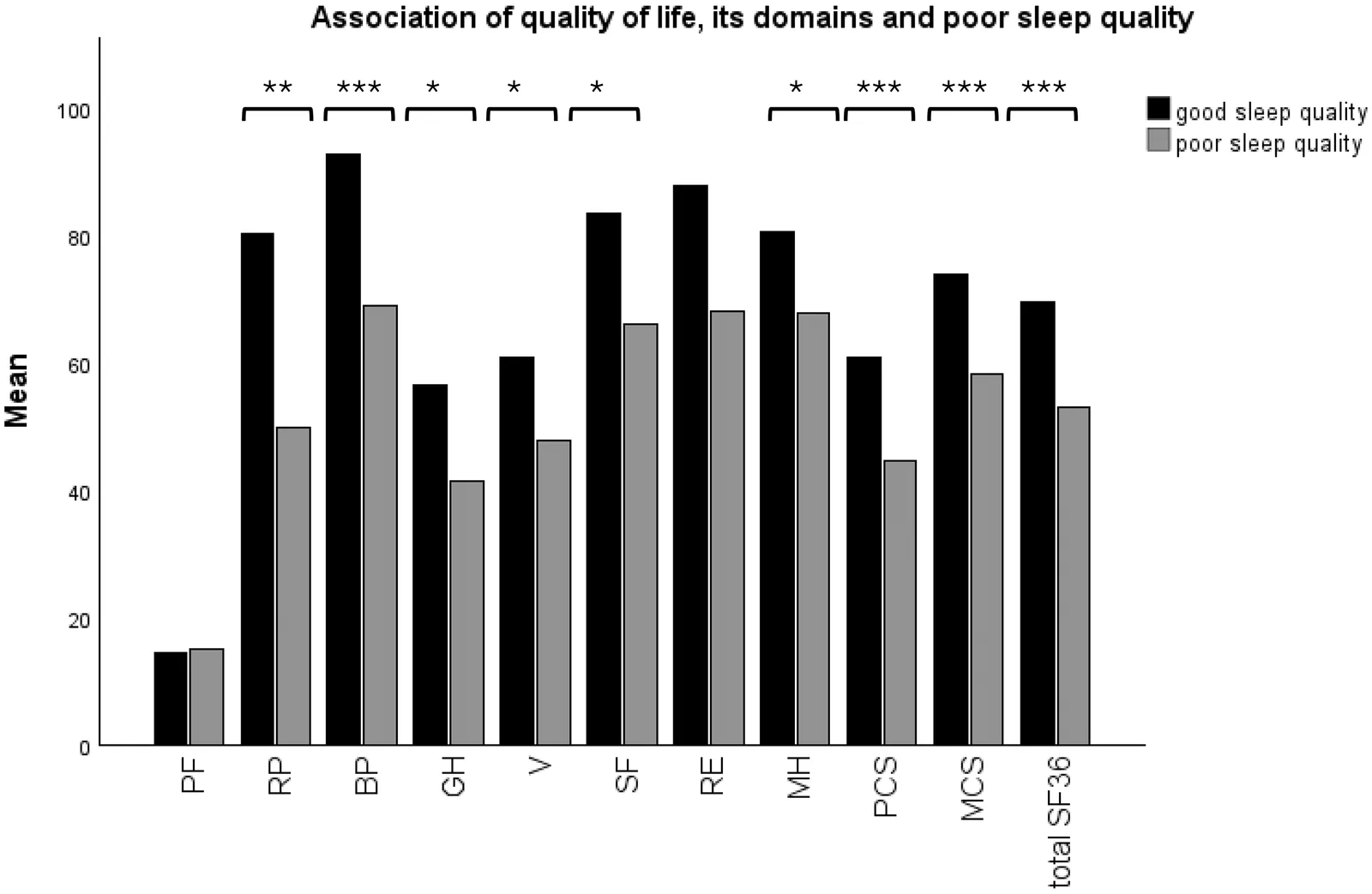
Association onf quality of life, its domains and poor sleep quality.

SMA patients classified as poor sleepers by the PSQI had lower scores across all QoL domains and in the total SF-36 score (53.2 ± 15.4 vs. 69.6 ± 12.3, p < 0.001), except for Physical Functioning and Role-Emotional, compared to those with good sleep quality (Figure 1).

PF – physical functioning, RP – role-physical, BP – bodily pain, GH – general health, V – vitality, SF – social functioning, RE – role-emotional, MH – mental health, PCS – physical component score, MCS – mental component score, SF-36–36-item short form survey; *—p < 0.05; **—p < 0.01; ***—p < 0.001.

#### One-year follow-up of sleep quality and excessive daytime sleepiness in included SMA

Follow-up data from 30 SMA patients were available. At the one-year follow-up, 43.3% of patients were classified as poor sleepers according to the PSQI. We observed no statistically significant differences in global PSQI score, except for a mild worsening in the sleep duration domain (0.3 ± 0.9 vs. 0.6 ± 0.9, p = 0.046).

Mean ESS score worsened over one year (4.0 ± 4.1 vs. 5.3 ± 4.6, p = 0.036). At the one-year follow-up, excessive daytime sleepiness was present in 13.3% of patients.

## Discussion

This study evaluated subjective sleep quality and excessive daytime sleepiness in adults with SMA, and their associations with QoL, depression, and fatigue. The study also provides novel insights by assessing, for the first time, the presence and severity of RLS in this population, conducting a comparison with an age- and sex-matched HC group, and presenting a one-year longitudinal follow-up of sleep quality in adults with SMA.

Compared to age- and sex-matched HC, SMA patients showed significantly poorer sleep efficiency and overall sleep quality. Our findings contrast with those of Wennberg et al., who also used the PSQI to assess sleep quality in 21 adult SMA patients and 23 matched controls, but reported no differences between groups across any sleep parameters.^9^ One possible reason for this discrepancy could be differences in disease severity, as the SMA patients in the Wennberg study had higher functional scores (mean HFMSE 37.0 ± 6.1; RULM 30.2 ± 8.0), indicating a milder disease phenotype. In our study, over half of the SMA patients were classified as poor sleepers, which aligns with the findings of Baldini et al., who reported that 60% of their SMA cohort experienced poor sleep quality.^28^ However, Baldini et al. did not include an age- and sex-matched control group, limiting direct comparisons. Our findings suggest that incorporating sleep quality assessment into the standard of care for adult SMA patients may be needed.

We observed that SMA patients classified as poor sleepers had higher BMI, more severe depressive symptoms, and greater daytime sleepiness. Crescimanno et al. speculated that elevated BMI may be a risk factor for obstructive sleep events in SMA, which could help explain the observed link between PSQI scores and BMI in our study.^10^ Previously, we reported a higher prevalence of metabolic syndrome in our adult SMA cohort compared to the general population, with central obesity being the most common component.^29^ This metabolic profile may contribute to impaired sleep quality and, in turn, negatively affect overall QoL in adults with SMA. Notably, the association between BMI and sleep quality appears to be SMA-specific since no correlation was found in our HC group. The link between depression and poor sleep quality has been previously documented in SMA^10^ and general population.^30^ However, this relationship may be bidirectional.^31^ Tricyclic antidepressants are generally effective in improving sleep quality, increasing total sleep time, and reducing wake time after sleep onset. In contrast, SSRIs have been shown to have the opposite effect.^32^ Given that up to one-quarter of SMA patients experience relevant symptoms of depression,^33^ tricyclic antidepressants may be a preferable option for treating depression in SMA patients with poor sleep quality.

The results of our study did not confirm previous observations suggesting that disease severity and functional status determine reduced sleep quality in adult SMA patients.^9,28^ In contrast, we found no association between motor function scores and subjective sleep quality, suggesting that the perception of sleep impairment might be highly individual. Furthermore, contrary to the results of Baldini et al., we found no differences in sleep quality between patients receiving nusinersen and risdiplam.

Our SMA patients classified as poor sleepers had lower scores across all QoL domains as well as in the total SF-36 score, except for Physical Functioning and Role-Emotional, compared to those with good sleep quality. This is in contrast to the results from the study of Baldini et al., who found no correlation between QoL (total SF-36 score) and sleep quality (PSQI score) in their study in 50 adult SMA patients.^28^ In the general population, poor sleep quality has been consistently linked to both physical and mental QoL.^4^ These findings emphasize the importance of incorporating sleep quality assessment into the standard of care for adults with SMA, as a critical factor in maintaining their overall QoL.

RLS was present in 11% of our SMA cohort. Due to the small sample size, we were only able to conduct descriptive statistics. Notably, this prevalence is significantly higher than the overall pooled prevalence of RLS in the general adult population, which is approximately 3%.^34^ Since the presence of RLS increases with age^34^ and SMA patients are typically young, this finding is particularly intriguing. However, it is essential to interpret these findings with caution, as our cohort of SMA patients was too small to support simple generalization. Future studies in larger cohorts of adult SMA patients are necessary to investigate this further. The prevalence of RLS in ALS, another motor neuron disease, has been reported to vary widely, ranging from 14.6% to 25%.^12^

The main limitation of our study is the relatively small number of individuals due to the single center design of the study. This limits the generalizability of our findings and highlights the need for further research. Prospective and multi-center studies involving larger numbers of individuals are necessary to fully understand the factors associated with sleep quality and the prevalence and significance of RLS in adult SMA. Another important limitation is the absence of objective sleep measures, such as polysomnography or cardiorespiratory polygraphy, and blood gas analysis, which would have enabled the detection of sleep-disordered breathing events, the most frequent cause of insomnia and excessive daytime sleepiness in neuromuscular diseases.^35^ Given the particular respiratory involvement in SMA,^1^ sleep-disordered breathing should be carefully considered in clinical follow-up. Future studies should aim to address these limitations and provide a more comprehensive understanding of sleep quality in adult SMA patients.

In conclusion, poor sleep quality is common in adults with SMA. Current international and German guidelines do not adequately address the importance of sleep quality in SMA.^36–38^ Given that sleep-related complaints are likely multifactorial, an individualized diagnostic and therapeutic approach may be required. Incorporation of a holistic approach to sleep-related complaints into the routine clinical care of adults with SMA is warranted.

## Abbreviations

ALSFRS-R: ALS Functional Rating Scale–Revised
BDI: Beck Depression Inventory
BMI: body mass index
ESS: Epworth Sleepiness Scale
FEV1: forced expiratory volume in one second
FSS: Fatigue Severity Scale
FVC: forced vital capacity
GH: general health
HC: healthy controls
HFMSE: Hammersmith Functional Motor Scale Expanded
MCS: mental composite score
MH: mental health
PCS: physical composite score
PEF: peak expiratory flow
PF: physical functioning
PSQI: Pittsburgh Sleep Quality Inventory
QoL: quality of life
RE: role emotional
RLS: restless legs syndrome
RP: role physical
RULM: Revised Upper Limb Module
SF: social functioning
SF-36: The 36-Item Short Form Health Survey
SMA: spinal muscular atrophy
SMAFRS: Spinal Muscular Atrophy Functional Rating Scale
*SMN2*: survival of motor neuron 2 gene
VT: vitality
